# The mitochondria-targeting compound PTC299 enhances megakaryocyte and platelet production

**DOI:** 10.1093/stcltm/szag035

**Published:** 2026-06-27

**Authors:** Xiaoli Wang, Meijuan Xia, Yifei Cai, Ziqi Huo, Yao Zhong, Pei Su, Cuicui Liu, Hongtao Wang, Xiangchun Shen, Fei Wang, Jianwei Xu, Jiaxi Zhou

**Affiliations:** School of Pharmacy, Guizhou Medical University, Gui'an New District, Guiyang 561113, China; State Key Laboratory of Experimental Hematology, National Clinical Research Center for Blood Diseases, Haihe Laboratory of Cell Ecosystem, Institute of Hematology & Blood Diseases Hospital, Chinese Academy of Medical Sciences & Peking Union Medical College, Tianjin 300020, China; Tianjin Institutes of Health Science, Tianjin 301600, China; Center for Tissue Engineering and Stem Cell Research, Guizhou Medical University, Gui'an New District, Guiyang 561113, China; State Key Laboratory of Experimental Hematology, National Clinical Research Center for Blood Diseases, Haihe Laboratory of Cell Ecosystem, Institute of Hematology & Blood Diseases Hospital, Chinese Academy of Medical Sciences & Peking Union Medical College, Tianjin 300020, China; Tianjin Institutes of Health Science, Tianjin 301600, China; State Key Laboratory of Experimental Hematology, National Clinical Research Center for Blood Diseases, Haihe Laboratory of Cell Ecosystem, Institute of Hematology & Blood Diseases Hospital, Chinese Academy of Medical Sciences & Peking Union Medical College, Tianjin 300020, China; Tianjin Institutes of Health Science, Tianjin 301600, China; State Key Laboratory of Experimental Hematology, National Clinical Research Center for Blood Diseases, Haihe Laboratory of Cell Ecosystem, Institute of Hematology & Blood Diseases Hospital, Chinese Academy of Medical Sciences & Peking Union Medical College, Tianjin 300020, China; Tianjin Institutes of Health Science, Tianjin 301600, China; State Key Laboratory of Experimental Hematology, National Clinical Research Center for Blood Diseases, Haihe Laboratory of Cell Ecosystem, Institute of Hematology & Blood Diseases Hospital, Chinese Academy of Medical Sciences & Peking Union Medical College, Tianjin 300020, China; Tianjin Institutes of Health Science, Tianjin 301600, China; State Key Laboratory of Experimental Hematology, National Clinical Research Center for Blood Diseases, Haihe Laboratory of Cell Ecosystem, Institute of Hematology & Blood Diseases Hospital, Chinese Academy of Medical Sciences & Peking Union Medical College, Tianjin 300020, China; Tianjin Institutes of Health Science, Tianjin 301600, China; State Key Laboratory of Experimental Hematology, National Clinical Research Center for Blood Diseases, Haihe Laboratory of Cell Ecosystem, Institute of Hematology & Blood Diseases Hospital, Chinese Academy of Medical Sciences & Peking Union Medical College, Tianjin 300020, China; Tianjin Institutes of Health Science, Tianjin 301600, China; State Key Laboratory of Experimental Hematology, National Clinical Research Center for Blood Diseases, Haihe Laboratory of Cell Ecosystem, Institute of Hematology & Blood Diseases Hospital, Chinese Academy of Medical Sciences & Peking Union Medical College, Tianjin 300020, China; Tianjin Institutes of Health Science, Tianjin 301600, China; School of Pharmacy, Guizhou Medical University, Gui'an New District, Guiyang 561113, China; HaemoCure Inc, Tianjin 300459, China; School of Pharmacy, Guizhou Medical University, Gui'an New District, Guiyang 561113, China; Center for Tissue Engineering and Stem Cell Research, Guizhou Medical University, Gui'an New District, Guiyang 561113, China; School of Pharmacy, Guizhou Medical University, Gui'an New District, Guiyang 561113, China; State Key Laboratory of Experimental Hematology, National Clinical Research Center for Blood Diseases, Haihe Laboratory of Cell Ecosystem, Institute of Hematology & Blood Diseases Hospital, Chinese Academy of Medical Sciences & Peking Union Medical College, Tianjin 300020, China; Tianjin Institutes of Health Science, Tianjin 301600, China

**Keywords:** PTC299, megakaryocyte, platelets, thrombocytopenia, DHODH, mitochondria

## Abstract

***Backgroud:*** Thrombocytopenia is a common complication of various clinical conditions, resulting from impaired megakaryocyte function, reduced platelet production, or excessive platelet destruction. Current treatments, including platelet transfusions and thrombopoietin receptor agonists, are limited by platelet supply constraints and risks such as thrombotic complications. Emerging research highlights the role of mitochondrial-related biological processes or components in thrombopoiesis, yet targeted therapeutics remain scarce.

***Methods:*** In this study, we investigated mitochondria-targeted compounds for their potential to enhance megakaryocyte and platelet productionusing a fetal liver megakaryocyte differentiation and platelet culture system, and explored their effects on *in vivo* megakaryocyte and platelet production using a radiation damage-induced thrombocytopenia mouse model.

***Results:*** Our findings identify PTC299, a dihydroorotate dehydrogenase and VEGFA mRNA translation inhibitor, as a promising pro-plateletogenic agent. PTC299 not only enhances megakaryocyte and platelet production *in vitro* but also accelerates their recovery in a mouse model of radiation-induced thrombocytopenia. Additionally, PTC299 alleviates irradiation-induced splenomegaly.

***Conclusion:*** PTC299 promotes megakaryocyte differentiation and platelet generation both *in vitro* and *in vivo*, demonstrating potential values for thrombocytopenia treatment and platelet regeneration.

## Graphical Abstract

**Graphical Abstract:**
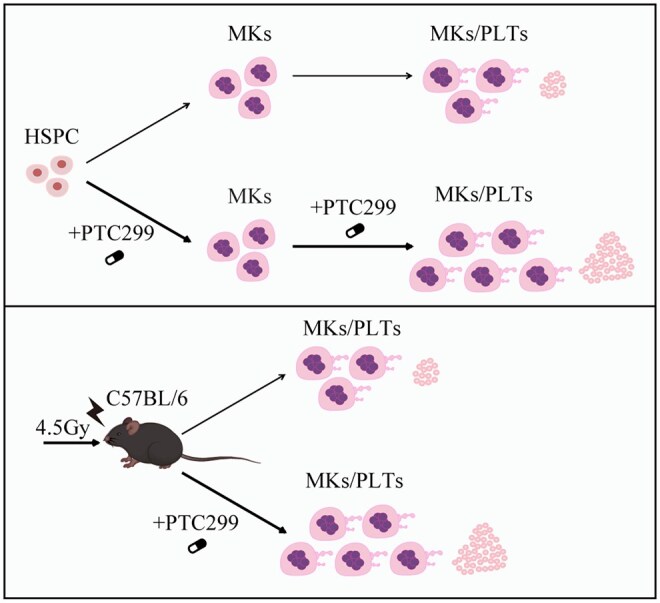

Significance statement The limitations of current thrombocytopenia therapies underscore the need for novel strategies to enhance platelet production. In this study, we identified PTC299 as a novel pharmacological agent that stimulates both megakaryopoiesis and thrombopoiesis. PTC299 significantly enhances megakaryocyte and platelet production *in vitro*, offering potential for large-scale platelet manufacturing. Moreover, PTC299 markedly accelerated the recovery of megakaryocytes and platelets in a mouse model of radiation-induced thrombocytopenia, highlighting its potential for *in vivo* therapeutic applications. Overall, PTC299 demonstrates potential values for thrombocytopenia treatment and platelet regeneration.

## Introduction

Megakaryocytes are rare, large, terminally differentiated blood cells within the hematopoietic system, primarily responsible for platelet production.^1^ During embryonic development, these cells are found in the yolk sac and liver,^2–4^ while in adulthood, the bone marrow serves as the primary site of thrombopoiesis.^5^^,^^6^ Megakaryocyte development, maturation, and platelet production are tightly regulated processes influenced by multiple transcription factors and cytokines.^7^ For instance, GATA1, a key transcription factor in megakaryocyte and erythroid lineage commitment, promotes megakaryocyte differentiation while suppressing myeloid cell fate.^8–10^ RUNX1 plays a crucial role in megakaryocyte differentiation and regulates the expression of essential platelet production genes, including NF-E2, which directly controls thrombopoiesis.^11–13^ Additionally, MEIS1 is critical for both megakaryopoiesis and thrombopoiesis from human pluripotent stem cells (hPSCs).^14^ At the cytokine level, thrombopoietin (TPO), stem cell factor (SCF), and interleukin-6 (IL-6) drive megakaryocyte proliferation and differentiation through distinct signaling pathways, collectively ensuring efficient platelet production.^15–18^

The importance of megakaryopoiesis and thrombopoiesis is also evident in a variety of diseases where these processes are defective.^19^ Thrombocytopenia, a condition characterized by low platelet counts, is a frequent complication of various clinical conditions, such as hematologic disorders,^20^ autoimmune diseases,^21^ infections,^22^ and chemotherapy.^23^ Impaired megakaryocyte differentiation and thrombopoiesis are key contributing factors to thrombocytopenia, as the production of platelets is closely tied to the proper maturation and function of megakaryocytes.^7^ Current treatment options, including corticosteroids,^24^ TPO receptor agonists,^25^ and rituximab,^26^ have demonstrated efficacy in managing thrombocytopenia. However, these treatments are not without limitations. For example, TPO receptor agonists may lose their effectiveness over time, and prolonged use can increase the risk of thrombotic events, which poses a significant concern.^27^ Moreover, corticosteroids and rituximab, while useful, may have side effects that complicate their long-term use. Given these challenges, identifying new targets or mechanisms regulating megakaryocyte differentiation and platelet production will provide new insights into the treatment of thrombocytopenia.

Mitochondria, as central regulators of cellular energy and redox metabolism, play pivotal roles in cell signaling and apoptosis pathways.^28^ Recent studies have shown that mitochondrial fission and reactive oxygen species (ROS) generated by mitochondrial genes can trigger thrombopoiesis in mature megakaryocytes.^29^ Additionally, the endogenous hormone arginine vasopressin (AVP) facilitates proplatelet formation and platelet release by phosphorylating Akt, which regulates mitochondrial metabolism, leading to a rapid surge in peripheral platelet levels.^30^ Furthermore, deficiencies in platelet-specific mitochondrial RNA metabolism (e.g., ELAC2 and PTCD1) or protein synthesis (e.g., MTIF3),^31^ as well as defects in megakaryocyte/platelet-specific mitochondrial membrane kinase AGK, can all lead to thrombocytopenia.^32^ These findings suggest that mitochondria-related metabolic processes or protein or RNA components are critical targets for modulating platelet production in megakaryocytes. However, the specific mitochondrial targets involved in regulating megakaryocyte or platelet production have not yet been adequately explored.

In this study, we sought to address this gap by screening compounds from a mitochondria-targeting library that influence reactive oxygen species, mitochondrial metabolism, autophagy, monoamine oxidase activity, pyruvate dehydrogenase kinase (PDHK), ATP synthase, and dihydroorotate dehydrogenase (DHODH). Using an *in vitro* megakaryocyte differentiation system with fetal liver-derived cells, we identified compounds that promote megakaryocyte differentiation and platelet production. The effects of these compounds on megakaryocyte differentiation, maturation, and platelet production were further examined in detail. Additionally, their efficacy was evaluated *in vivo* using a mouse model of thrombocytopenia induced by sublethal doses of ionizing radiation (IR). This research provides innovative insights into targeting mitochondrial components to enhance megakaryocyte differentiation and platelet production, offering potential advancements in thrombocytopenia management.

## Methods

### Drugs, reagents, and antibodies

PTC299 (HY-124593, purity > 99.5%), Teriflunomide, Brequinar, and the mitochondrial targeting compound library were purchased from MedChemexpress Biotechnology, Inc. Mouse VEGF-A ELISA kit was purchased from Sangon Biotech, Inc. Apoptosis detection kit was purchased from YEASEN, Inc. Phosphatase inhibitor cocktail, ROS and mitochondrial membrane potential detection kit were purchased from Beyotime, Inc. Counting beads was purchased from BD Bioscience, USA. DMEM basal medium and fetal bovine serum (FBS) were purchased from Gibco, Inc. Antibodies 780 anti-mouse CD11b, APC-eFluor™ 780 anti-mouse Ly6G/Ly6C, APC-eFluor™ 780 anti-mouse CD45R/B220, and APC-eFluor™ 780 anti-mouse Ter119 were purchased from eBioscience, USA. FITC anti-mCD41 and APC anti-hCD41 were purchased from BD Bioscience, USA. The antibodies APC anti-mouse CD117 (c-kit), PE-Cy7 anti-mouse Sca-1, APC anti-mCD42d, PE anti-mCD41, Per-Cy5.5 anti-mCD42d, PE-Cy7 anti-mCD61 antibodies, and Hoechst 33342 were purchased from BioLegend, USA.

### Mice

All animal experiments were approved by the Animal Ethics Committee of the State Key Laboratory of Experimental Hematology (SKLEH), Institute of Hematology and Blood Diseases Hospital, Chinese Academy of Medical Sciences (ethical approval number: IHCAMS-DWLL-NSFC2024137-1) and conducted in accordance with the Guide for the Care and Use of Laboratory Animals. Eight-week-old female SPF-grade C57BL/6 mice, weighing approximately 18 g, were purchased from Huafukang Company, Beijing, China. The mice were housed in a barrier system at the Cellular Ecology Haihe Laboratory Animal Center, with free access to food and water. They were maintained under suitable conditions (temperature 25 ± 2 °C, humidity 55% ± 5%, 12-h light-dark cycle).

### Megakaryocyte induction from mouse fetal liver progenitor cells

Single-cell suspension of E13.5-E15.5 fetal liver was obtained by grinding, red blood cells (RBC) were lysed, and then resuspended in PBE buffer (PBS + 2% FBS + 0.04% 0.5M EDTA). Next, the cells were incubated with a Lineage Series Antibody cocktail [including CD3, CD11b, CD45R/B220, CD117 (c-kit), and Ter119] for 30 min at 4 °C. Lin^-^ c-kit^+^ HSPCs were obtained by sorting from BD FACSAria Fusion. The fetal liver nucleated cells or fetal liver HSPCs were resuspended in DMEM medium supplemented with 10% FBS, 1% penicillin-streptomycin-amphotericin B mixture, and 50 ng/mL mTPO. Cells were seeded in 24-well plates or 8-well chamber slides at a density of 5 × 10^4^ cells per well, designated as day 0 (D0). The cultures were maintained in a cell culture incubator (Thermo Fisher Scientific, USA) maintained 5% CO_2_ at 37 °C. On day 1 (D1), different concentrations of PTC299 (0.5, 1, 2 μm) or other mitochondrial targeting compounds were added to the cultures. The proportion of CD41^+^ or CD41^+^CD42d^+^ megakaryocytes was assessed using BD LSRFortessa X-20 on day 3 to 5 (D3-D5).

### Platelet production from mouse fetal liver megakaryocytes

CD41 and CD42d antibodies were added to the single-cell suspension of fetal liver according to experimental requirements and incubated at 4 °C for 30 min. CD41^+^CD42d^+^ megakaryocytes were sorted using FACSAriaIII sorting flow cytometer (BD, USA), replacing the nozzle with a 100 μm nozzle and adjusting the flow sorting parameters accordingly. The sorted megakaryocytes were centrifuged at 350*g* for 10 min and resuspended in StemSpan TMSFEM medium supplemented with 25 ng/mL mTPO, 25 ng/mL mSCF, 10 ng/mL mIL6, 10 ng/mL mIL9, 5 U/mL sodium heparin, and 5 ng/mL Y-27632. Cells were seeded at a density of 2500 per well in 24-well plates with different concentrations of PTC299 (0.5, 1, 2 μm) and incubated at 37 °C in a 5% CO_2_ incubator. The status of megakaryocytes was monitored daily to assess the initiation of platelet production. Platelet-producing morphology of megakaryocyte was analyzed via immunofluorescence by Andor Dragonfly 200 confocal microscope, and after 48 h of incubation, the platelets present in the culture supernatant were quantified by flow cytometry to determine their proportion and number.

### Measurement of megakaryocyte and platelet production ratios *in vitro*

Cells were collected after *in vitro* megakaryocyte-induced differentiation culture and labeled with CD41, CD42d, and Hoechst 33342 (1:300). The samples were incubated at room temperature, protected from light, for 30 min. Afterward, 1 mL of PBE was added for elution, followed by centrifugation at 200*g* for 5 min. The cells were resuspended in 200 µL of PBE and analyzed using a BD LSRFortessa X-20 flow cytometer. The proportion of CD41^+^ or CD41^+^CD42d^+^ megakaryocytes was measured, and the ploidy distribution of megakaryocytes was examined.

For platelet analysis, the culture medium supernatant after *in vitro* platelet generation induction was collected. PE anti-mCD41, Per-Cy5.5 anti-mCD42d, and PE-Cy7 anti-mCD61 antibodies were added at a volume ratio of 1:500, and Hoechst 33342 was added at a volume ratio of 1:300. The mixture was incubated at 37 °C for 30 min. Following incubation, 1 mL of PBE was added to wash the antibodies, and the sample was centrifuged at 3500 rpm for 5 min. The cells were then resuspended in 200 µL of PBE. The ratio of CD41^+^CD61^+^ or CD42d^+^CD61^+^ platelets was detected using a BD LSRFortessa X-20 Analytical Flow Cytometer.

### VEGFA content detection

VEGF-A content was measured after 3 days in culture. Approximately 3-4 million cells were harvested, washed 3 times with cold PBS, and resuspended in a cold PBS solution supplemented with a protease and phosphatase inhibitor cocktail (P1045, Beyotime). To lyse the cells, the suspension was subjected to 3 freeze-thaw cycles. Each cycle consisted of freezing at −80 °C for 20 min, followed by thawing at room temperature for 20 min, with vigorous vortexing before the next cycle. The homogenate was centrifuged at 1500*g* for 10 min at 4 °C. The clarified supernatant was then analyzed for VEGF-A levels using a specific mouse VEGF-A ELISA kit (D721156, Sangon Biotech), following the manufacturer’s protocol.

### Apoptosis, ROS, and JC-1 detection

On day 3 of *in vitro* culture, cells were harvested and analyzed by flow cytometry for surface marker expression and functional assays. Briefly, cells were stained with a panel of antibodies including PE-Cy7-lineage, APC-c-Kit, APC-Cy7-CD41, and PerCP-Cy5.5-CD42d. Concurrently, apoptosis was detected using a specific kit (40302ES60, YEASEN), reactive oxygen species (ROS) levels were measured with kit S0033 (Beyotime), and mitochondrial membrane potential was assessed using kit C2006 (Beyotime), all performed according to the manufacturers’ instructions. Prior to analysis, DAPI was added to exclude nonviable cells.

### Platelet production from human-induced pluripotent stem cells

Hematopoietic progenitor cells were generated from the hiPSC hematopoietic differentiation system as previously described.^14^ 5 × 10^5^ cells/mL cells were cultured in StemSpan SFEM serum-free medium supplemented with 50 ng/mL TPO, 20 ng/mL SCF, 15 ng/mL IL-3, 15 ng/mL IL-6, and 20 ng/mL IL-11 for 9 days. Fresh medium was used every 3 days. In addition, 75 nM StemRegenin 1 was added in the first 3 days, and 5 μm Y-27632, 15 µM GM6001, and 100 nM Ruxolitinib was added after 3 days of culture. PTC299 was applied during the day 0-day 9 differentiation window. After 9 days, platelets were enriched using a BSA gradient method.^33^ Cells were then labeled with APC-conjugated anti-human CD41 antibody at room temperature for 30 min. The proportion of CD41^+^ platelets was analyzed by flow cytometry, and absolute platelet counts were determined using counting beads (340334, BD Trucount™ Tubes).

### X-ray–induced thrombocytopenia models

After 1 week of acclimatization, C57BL/6 mice were randomly divided into 3 groups: the IR group (X-rays), the PTC299 low-dose group (X-rays + 3 mg/kg PTC299), and the PTC299 high-dose group (X-rays + 4.5 mg/kg PTC299). The mice were irradiated with 4.5 Gy of X-rays to establish a thrombocytopenia model.

For administration, mice in the low and high-dose PTC299 groups received daily intraperitoneal injections of 3 and 4.5 mg/kg PTC299, respectively, starting on the ninth day after irradiation. Mice in the IR group were given an equal volume of PBS by intraperitoneal injection.

Blood samples were collected from the tail tip into 1.5 mL anticoagulant tubes. After mixing, 10 µL of whole blood was added to 240 µL of PBS to dilute the sample (1:25). Basic blood cell parameters, including platelet counts, mean platelet volume (MPV), platelet distribution width (PDW), and RBC and leukocyte counts, were measured using a Myriad automatic hemocytometer.

### Measurement of megakaryocytes and hematopoietic stem/progenitor cells in the thrombocytopenic model mice

To analyze HSPC in the bone marrow of thrombocytopenic mice, bone marrow cells were collected 13 days after modeling (4 days after PTC299 administration) and labeled with a 1:100 volume ratio of APC-eFluor™ 780 anti-mouse CD3, APC-eFluor™ 780 anti-mouse CD11b, APC-eFluor™ 780 anti-mouse Ly6G/Ly6C, APC-eFluor™ 780 anti-mouse CD45R/B220, APC-eFluor™ 780 anti-mouse Ter119, APC anti-mouse CD117 (c-kit), and PE-Cy7 anti-mouse Sca-1 for 30 min at 4 °C, protected from light. The percentage of HSPCs was detected by BD LSRFortessa X-20 analytical flow cytometry.

To analyze MK in the bone marrow of thrombocytopenic mice, FITC anti-mouse CD41 and APC anti-mouse CD42d antibodies were added to the bone marrow cells as described above at a volume ratio of 1:500. The cells were labeled at 4 °C for 30 min, protected from light. The ratio of MK was detected by BD LSRFortessa X-20 analytical flow cytometry.

### Immunofluorescence

To detect the effect of PTC299 on platelet production by megakaryocytes, PE anti-mouse CD41, APC anti-mouse CD42d antibodies, and Hoechst 33342 (1:100) were added to megakaryocyte cultures after 48 h of incubation at a dilution of 1:200. The cells were incubated for 30 min at room temperature, protected from light. The effect was then evaluated by imaging using the Andor Dragonfly 200 confocal microscope. Additionally, the effect of PTC299 on the morphology of megakaryocyte platelet production was assessed.

### Hematoxylin and eosin staining

Mouse femur and spleen tissues, collected 13 days after modeling (4 days after PTC299 administration), were separated and fixed in 10% formalin at room temperature for 24 h. After dehydration, paraffin embedding, and sectioning, the tissues were stained with hematoxylin and eosin (HE). The images were captured using a light microscope.

### Statistical analysis

One-way (Figures S1B, D, S2A, D, and S3B-G [see online supplementary material for a color version of these figures] and Figures 2C, E, H, 3C, D, 5, and 6) or two-way ANOVA (Figure 4A-F), followed by Dunnett’s *post hoc* test, were used to assess statistical significance among multiple treatment groups. Unpaired two-tailed *t*-tests were applied for all other comparisons (*P < .05; **P < .01; ***P < .001; ns, not significant), with a *P* value of < .05 considered statistically significant. All data are presented as mean ± standard deviation. Specific statistics and statistical significance are described in the figure legends. All data were analyzed using GraphPad Prism 8 and GraphPad Prism 10 software (versions 8.0.1 and 10.5.0).

## Results

### Several mitochondria-targeting compounds promote differentiation of fetal liver nucleated cells to CD41-positive megakaryocytes

**Figure 1.**
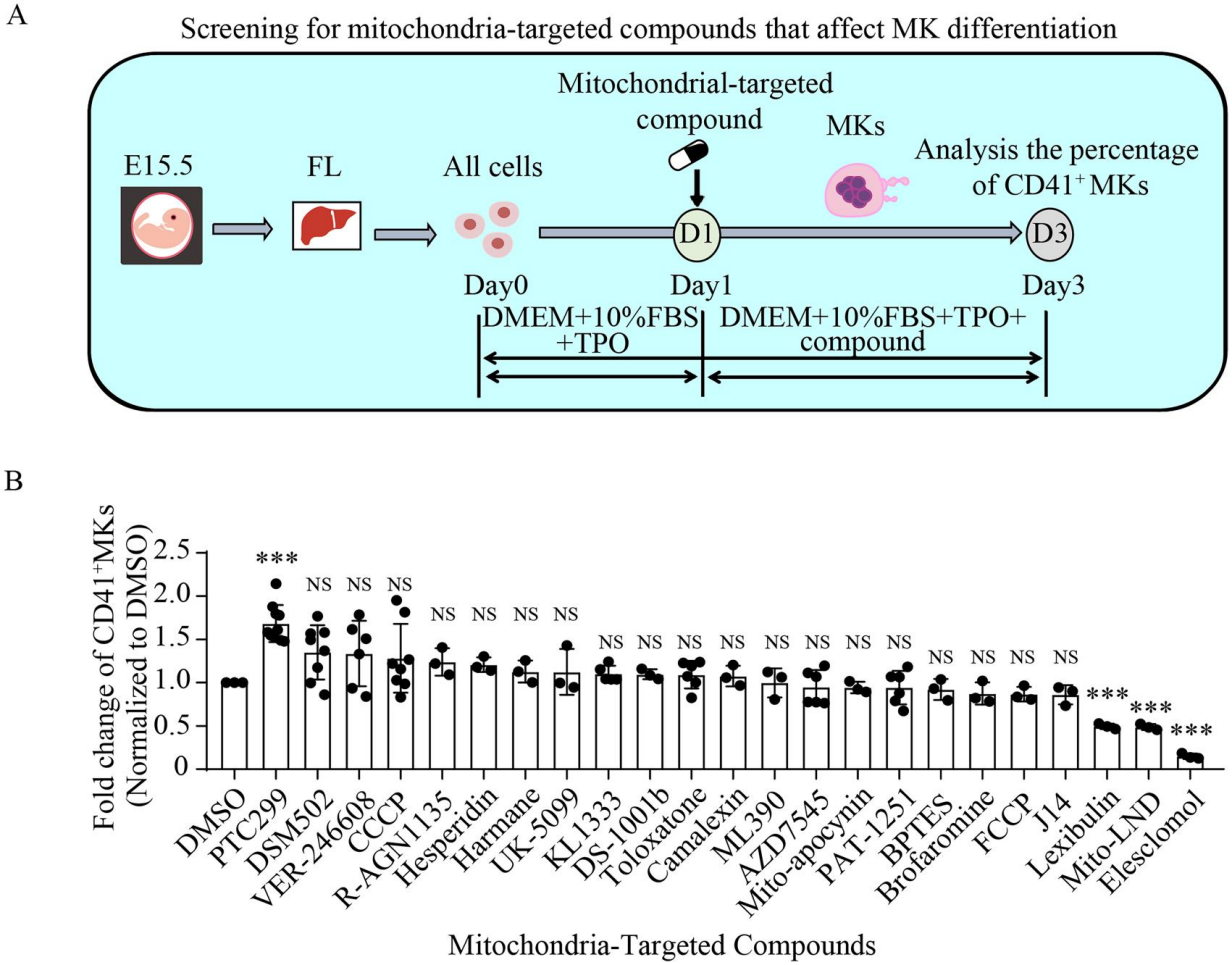
The identification of mitochondrial-targeted compounds that affect mouse megakaryocyte production. (A) Schematic diagram showing the culture system for screening mitochondria-targeting compounds that affect the differentiation of whole fetal liver nucleated cells to megakaryocytes in E15.5 mice. The complete list of examined compounds was shown in Table S1. (B) The effects of 23 mitochondria-targeting compounds on the proportion of fetal liver nucleated cells differentiated to produce CD41^+^ megakaryocytes compared with the control. Unpaired *t* test, ***P < .001. All values were normalized to the level (=1) of DMSO.

To screen for mitochondria-targeting compounds that promote megakaryopoiesis, we established an induction culture system for differentiating whole fetal liver nucleated cells into megakaryocytes. Whole fetal liver nucleated cells from E15.5 mice were cultured in DMEM medium supplemented with 10% FBS and 50 ng/mL mTPO. Mitochondria-targeting compounds were added on day 1 (D1), and the percentage of CD41-positive megakaryocytes was assessed by flow cytometry on day 3 (D3; Figure 1A). Using this system, we screened 23 mitochondria-targeting compounds and discovered that inhibitors targeting DHODH, such as PTC299 and DSM502, and the pyruvate dehydrogenase kinase (PDHK) inhibitor VER-246608, promoted the generation of CD41-positive megakaryocytes from fetal liver cells to varying extents (Figure 1B). Among these, PTC299 significantly promoted the differentiation of fetal liver nucleated cells into large cells (>30 μm; Figure S1A and B, see online supplementary material for a color version of this figure) and strongly enhanced CD41-positive megakaryocyte generation at a concentration of 1 and 5 μm (Figure1B and Figure S1C and D, see online supplementary material for a color version of this figure). In contrast, compounds inducing the production of reactive oxygen species, including Elesclomol, Mito-LND, and Lexibulin, markedly inhibited the generation of CD41-positive megakaryocytes in fetal liver cultures (Figure 1B).

### PTC299 promotes megakaryocyte differentiation and maturation of fetal liver HSPCs

PTC299 is recognized as a potent inhibitor of DHODH and an oral VEGFA mRNA translation inhibitor, selectively blocking VEGF protein synthesis in the post-transcriptional level.^34^ However, the role of DHODH and the specific effects of PTC299 in megakaryocyte differentiation remain unclear.

**Figure 2.**
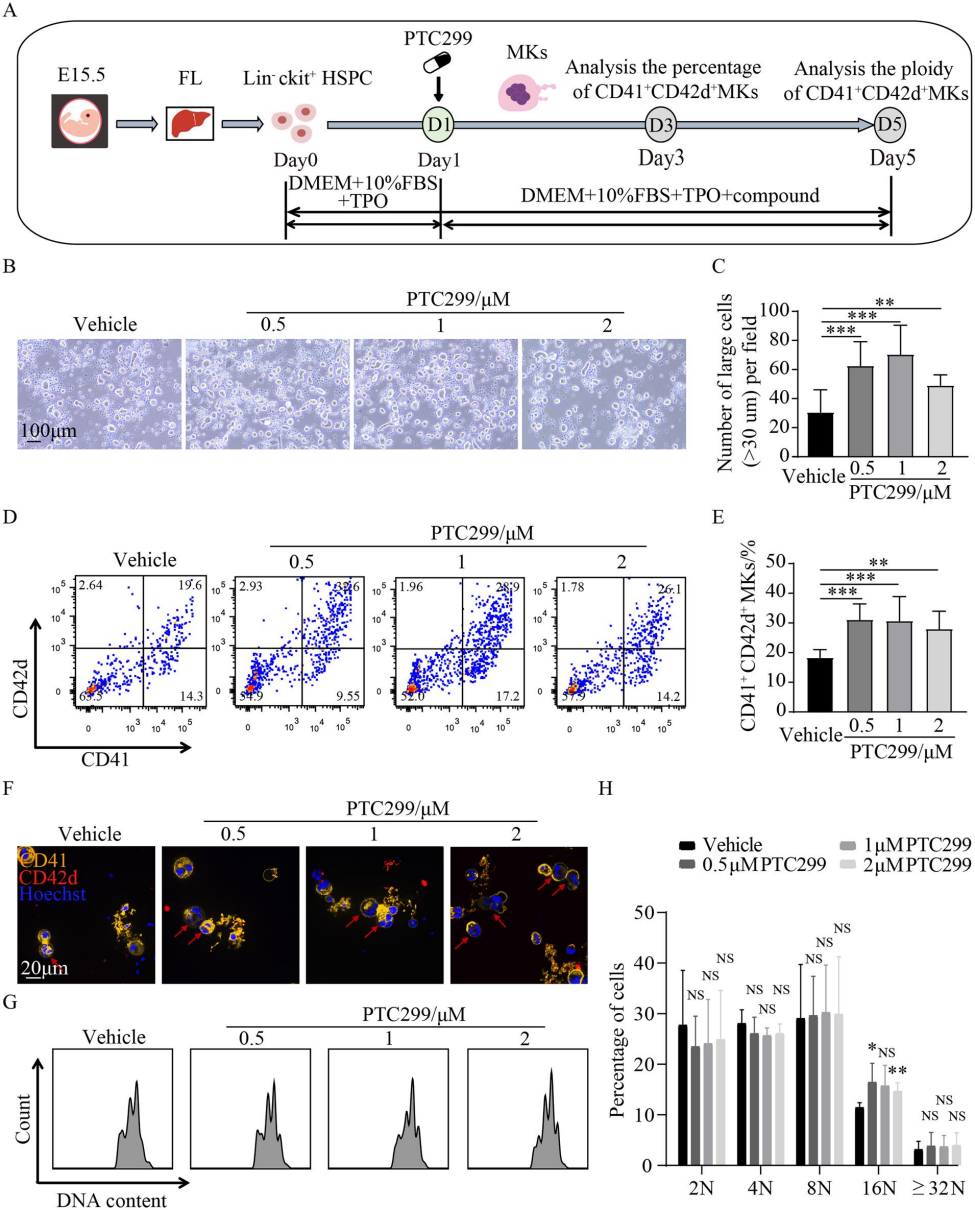
PTC299 promotes the differentiation of mouse fetal liver hematopoietic stem and progenitor cells into megakaryocytes. (A) Schematic diagram showing the culture system for differentiation of fetal liver hematopoietic stem progenitor cells (HSPC) to megakaryocytes in E13.5-E15.5 mice. (B) Representative photomicrographs of megakaryocyte differentiation on day 3 after the treatment of HSPCs with different concentrations of PTC299 (0.5, 1, and 2 μm) with scale bar = 100 μm. (C) Quantification of the number of large cells (>30 μm) per field on day 3 after the treatment of sorted HSPCs with different concentrations of PTC299 (0.5, 1, and 2 μm), in comparison with the control (vehicle only) group. One-way ANOVO followed by Dunnett’s *post hoc* test, **P < .01, ***P < .001. (D) Representative flow plots of HSPCs treated with different concentrations of PTC299 (0.5, 1, and 2 μm) on day 3. (E) Percentages of CD41^+^CD42d^+^ cells on day 3 after the treatment of HSPCs with different concentrations of PTC299 (0.5, 1, and 2 μm), in comparison with the control. One-way ANOVO followed by Dunnett’s *post hoc* test, **P < .01, ***P < .001, *n* = 3. (F) Representative immunofluorescence images of megakaryocytes on day 5 after the treatment of HSPCs with different concentrations of PTC299 (0.5, 1, and 2 μm). Scale bar = 20 μm, and the arrows indicate polyploid megakaryocytes. (G) Flow cytometry analysis measuring the ploidy of representative CD41^+^CD42d^+^ megakaryocytes on day 5 after the treatment of HSPCs with different concentrations of PTC299 (0.5, 1, and 2 μm). (H) Quantification of ploidy distribution of CD41^+^CD42d^+^ megakaryocytes on day 5 after the treatment of HSPCs with different concentrations of PTC299 (0.5, 1, and 2 μm). One-way ANOVO followed by Dunnett’s *post hoc* test, *P < .05, **P < .01, *n* = 6.

To address this, we established a directed differentiation culture system using mouse fetal liver hematopoietic stem progenitor cells (HSPCs) to investigate the impact of PTC299 on megakaryocyte differentiation. PTC299 was administered at varying concentrations (0.5, 1, and 2 μm) on day 1 (D1), and megakaryocyte generation was assessed on day 3 (D3; Figure 2A). The results revealed that all concentrations of PTC299 did not affect the number of fetal liver cells and the activity of megakaryocytes (Figure S2A and B, see online supplementary material for a color version of this figure), but it significantly promoted the differentiation of HSPCs into large cells (>30 μm; Figure 2B). Notably, the number of large cells generated with 0.5 and 1 μm PTC299 was approximately double that of the control group (Figure 2C).

To further validate this effect, we performed flow cytometry analysis, showing that 0.5, 1, and 2 μm PTC299 significantly increased the proportion of CD41^+^CD42d^+^ megakaryocytes compared to the control group, with an enhancement of approximately 1.5 times (Figure 2D and E). These findings indicate that PTC299 strongly promotes megakaryopoiesis.

PTC299 is both a DHODH inhibitor and a VEGFA mRNA translation inhibitor. To clarify the potential target of PTC299, we detected VEGFA levels after 3 days of culture using ELISA. After PTC299 treatment, the VEGFA level in cells did not change significantly (Figure S2C, see online supplementary material for a color version of this figure), suggesting that PTC299 does not promote megakaryocyte generation by affecting VEGFA. Moreover, similar to PTC299, the DHODH inhibitor Teriflunomide also significantly increased the proportion of CD41^+^CD42d^+^ megakaryocytes. In contrast, another DHODH inhibitor, Brequinar, exerted a comparatively weaker effect on megakaryocyte differentiation (Figure S2D, see online supplementary material for a color version of this figure), suggesting that PTC299 may promote megakaryocyte generation by inhibiting DHODH.

To further explore whether PTC299 also influences megakaryocyte maturation, we cultivated cells until day 5 and analyzed for morphological changes using immunofluorescence. At all tested concentrations, PTC299 enhanced nuclear lobation in megakaryocytes (Figure 2F), suggesting a role in promoting polyploidization. This observation was further confirmed by flow cytometric analysis of ploidy distribution, which demonstrated that PTC299 significantly increased the proportion of 16 N megakaryocytes on day 5 (Figure 2G and H).

Together, these findings demonstrate that PTC299 not only enhances the differentiation of megakaryocytes from fetal liver hematopoietic stem progenitor cells but also promotes their maturation. These results underscore the potential of PTC299 as a key regulator of megakaryocyte differentiation and maturation.

### PTC299 promotes platelet production of fetal liver megakaryocytes

We next asked whether PTC299 could also promote platelet production of the megakaryocytes derived from fetal liver hematopoietic stem progenitor cells. To address this, the platelet-producing morphology of the megakaryocytes was analyzed via immunofluorescence, and the proportion and number of platelets produced were assessed by flow cytometry, on the fifth day of cultivation. As expected, PTC299 significantly promoted proplatelet production from the megakaryocytes differentiated from fetal liver HSPCs, as shown by immunofluorescence (Figure S3A, see online supplementary material for a color version of this figure). Furthermore, the addition of PTC299 significantly increased the proportion and/or number of CD41^+^CD61^+^ and CD42d^+^CD61^+^ platelets (Figure S3B and C, see online supplementary material for a color version of this figure). Additionally, we found that Teriflunomide modestly promoted the generation of CD41^+^CD61^+^ and CD42d^+^CD61^+^ platelets, whereas Brequinar showed a comparatively weaker effect on both the proportion and the absolute number of platelets (Figure S3D-G, see online supplementary material for a color version of this figure).

**Figure 3.**
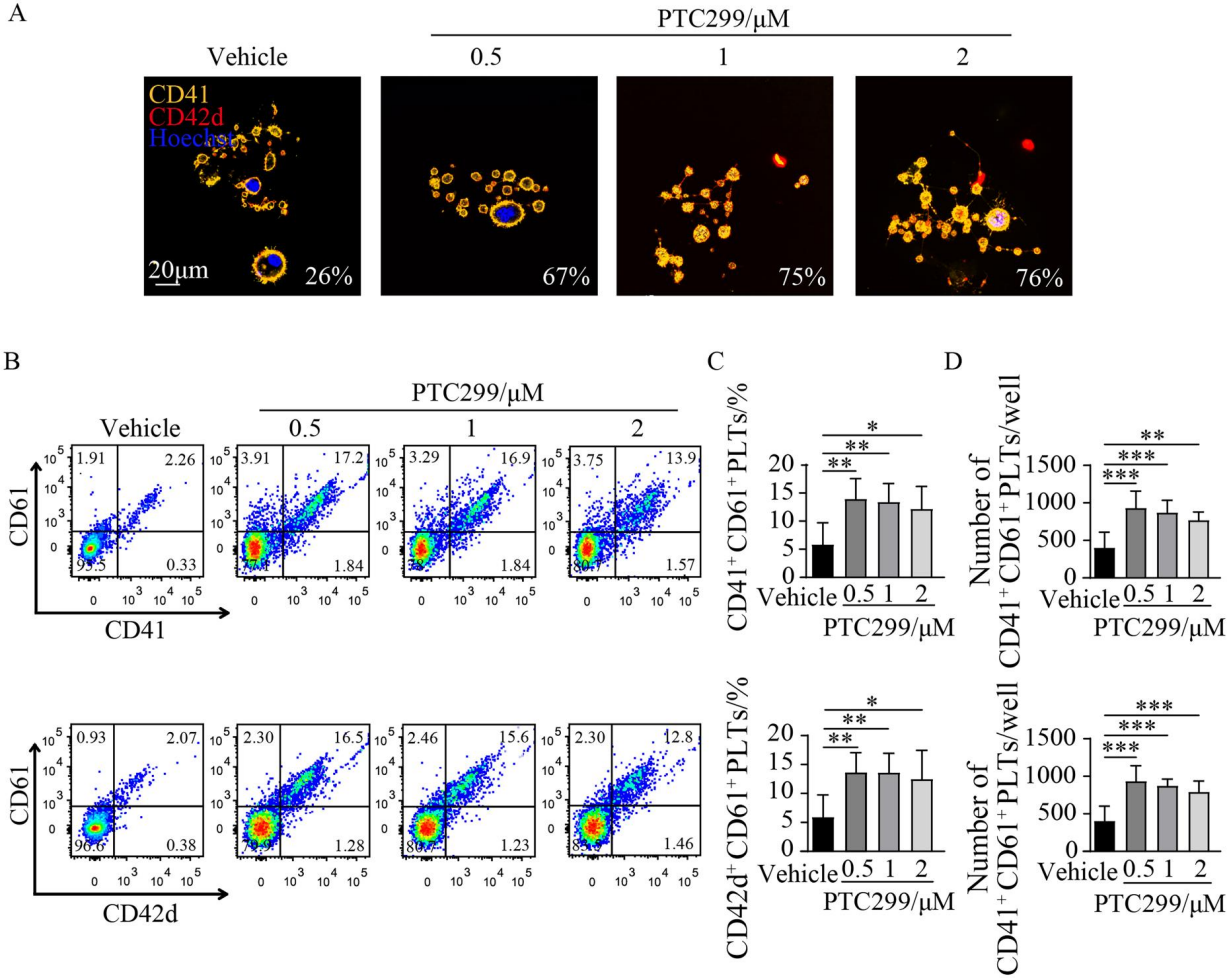
PTC299 promotes platelet production of mouse fetal liver megakaryocytes *in vitro*. (A) Immunofluorescence images of fetal liver proplatelet-forming megakaryocytes on day 2 after the treatment with different concentrations of PTC299 (0.5, 1, and 2 μm); scale bar = 20 μm. The number in the lower right corner of the picture indicates the proportion of megakaryocytes that produce proplatelets. (B) Representative flow cytometric analyses of CD41^+^CD61^+^ and CD42d^+^CD61^+^ platelets generated by fetal liver megakaryocytes at 48 h after the treatment with different concentrations of PTC299 (0.5, 1, and 2 μm). (C) Quantification of the proportion of CD41^+^CD61^+^ and CD42d^+^CD61^+^ platelets produced from fetal liver megakaryocytes 48 h after the treatment of different concentrations of PTC299 (0.5, 1, and 2 μm), in comparison with the control group. One-way ANOVO followed by Dunnett’s *post hoc* test, *P < .05, **P < .01, *n* = 3. (D) The numbers of CD41^+^CD61^+^ and CD42d^+^CD61^+^ platelets produced from fetal liver megakaryocytes 48 h after the treatment with different concentrations of PTC299 (0.5, 1, and 2 μm), in comparison with the control. One-way ANOVO followed by Dunnett’s *post hoc* test, **P < .01, ***P < .001, *n* = 3.

To further evaluate the effect of PTC299 on platelet production by primary fetal liver-derived megakaryocytes, we sorted fetal liver CD41^+^CD42d^+^ megakaryocytes and cultured them in Stemspan TMSFEM medium supplemented with cytokines. After 2 days of cultivation, platelet-producing morphology of the megakaryocytes was analyzed via immunofluorescence, and the proportion and number of platelets produced were assessed by flow cytometry. Immunofluorescence results showed that the number of megakaryocytes exhibiting platelet-producing morphology was significantly higher in the PTC299-treated group compared to the control group (Figure 3A). Flow cytometry analysis revealed that the addition of 0.5, 1, and 2 μm PTC299 significantly increased the ratio of CD41^+^CD61^+^ and CD42d^+^CD61^+^ platelets (Figure 3B and C) and approximately doubled the number of CD41^+^CD61^+^ and CD42d^+^CD61^+^ platelets produced (Figure 3D). These findings suggest that PTC299 also promotes platelet production of fetal liver megakaryocytes, highlighting its potential medicinal value in thrombopoiesis.

### PTC299 did not clearly enhance platelet output in the human-induced pluripotent stem cell–based system

To further evaluate whether PTC299 also promotes platelet production in humans, we examined its effects using a hiPSC-derived megakaryocyte differentiation and platelet generation platform. PTC299 was administered during the day 0-day 9 differentiation window, and platelet yield was assessed on day 9. We found that PTC299 significantly increased the percentage of CD41^+^ platelets but did not increase the absolute number of CD41^+^ platelets (Figure S4A and B, see online supplementary material for a color version of this figure). These results suggest that, in this hiPSC-based system, PTC299 does not clearly enhance platelet output. Therefore, the role of PTC299 in regulating human platelet production warrants further investigation.

### PTC299 expedites platelet recovery in mice with radiation-induced thrombocytopenia

**Figure 4.**
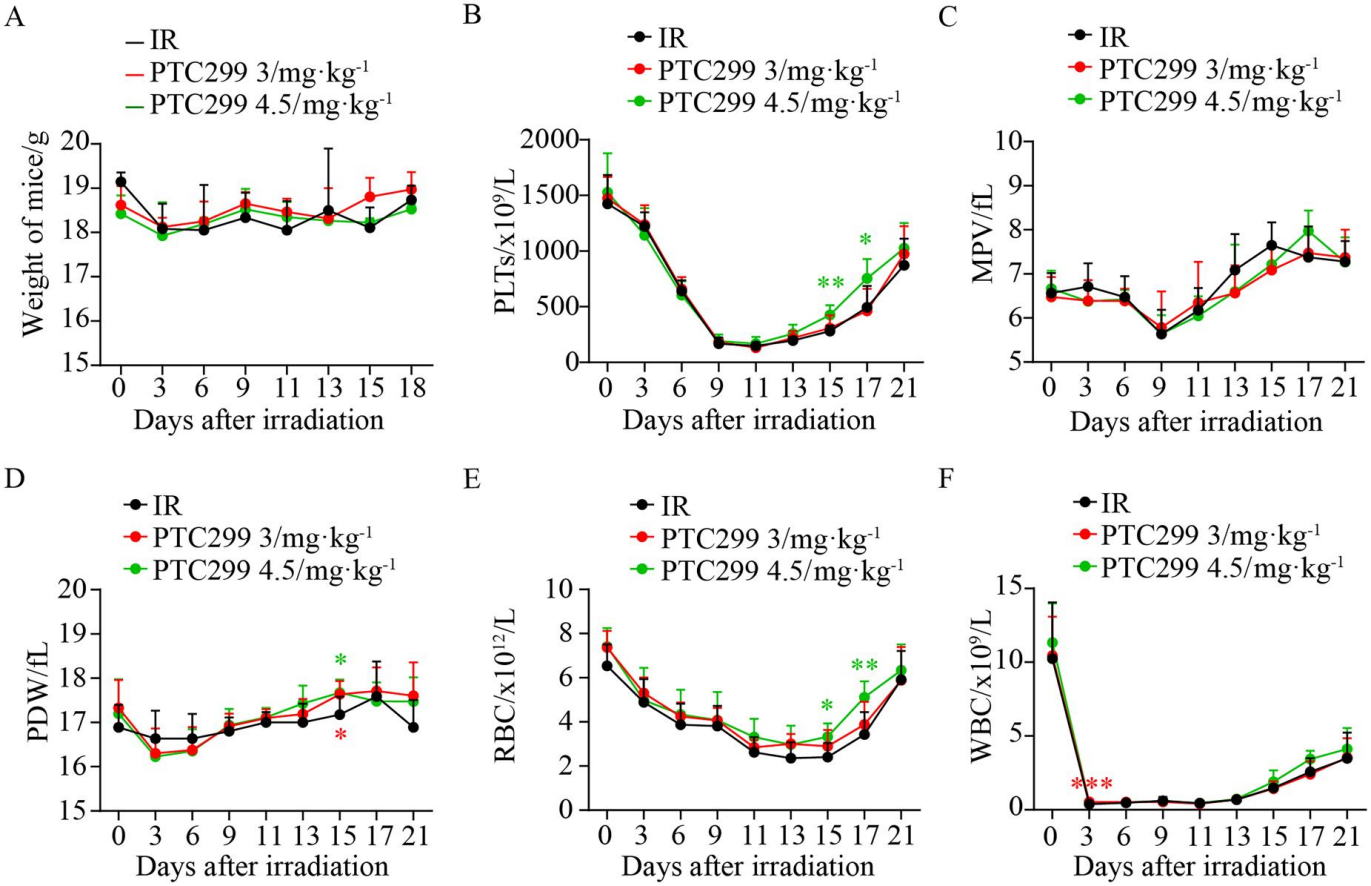
PTC299 expedites platelet restoration in radiation-induced thrombocytopenic mice. (A-H). Blood routine dynamics in a mouse model of X-ray-induced thrombocytopenia mice irradiated at 4.5 Gy and treated with PTC299 (3 or 4.5 mg/kg) or left untreated (IR control). (A) Body weights of the mice. Error bars indicate standard deviation, *n* = 3. (B) Peripheral blood platelet count, two-way ANOVO (mixed-effects model) followed by Dunnett’s *post hoc* test, *P < .05, **P < .01, *n* = 7-8. (C) Peripheral blood platelet mean volume (MPV), *n* = 7-8. (D) Peripheral blood platelet distribution width (PDW), two-way ANOVO (mixed-effects model) followed by Dunnett’s *post hoc* test, *P < .05, *n* = 7-8. (E) Peripheral blood red blood cell count, two-way ANOVO (mixed-effects model) followed by Dunnett’s *post hoc* test, *P < .05, **P < .01, *n* = 7-8. (F) Peripheral blood white blood cell count, two-way ANOVO (mixed-effects model) followed by Dunnett’s *post hoc* test, ***P < .001, *n* = 7-8.

Building upon the *in vitro* findings that PTC299 promotes megakaryocyte differentiation and platelet production, we further investigated its effects *in vivo* using a mouse model of thrombocytopenia induced by X-ray irradiation at 4.5 Gy. Following radiation exposure, peripheral blood platelet counts in mice sharply declined, reaching their lowest levels between day 9 and 11, before gradually recovering (Figure 4B).

To assess the impact of PTC299, mice in the experimental group received daily intraperitoneal injections of 3 or 4.5 mg/kg PTC299 starting on day 9, while control (irradiation only, denoted as IR) mice received equal amounts of PBS solvent (contain <2% DMSO). Body weights remained comparable between PTC299-treated and control mice (Figure 4A), and treated mice displayed normal appetite and overall condition, indicating no significant drug toxicity.

Regarding platelet recovery, mice administered 4.5 mg/kg PTC299 exhibited significantly higher platelet counts than the control group after 6 days of treatment (day 15; Figure 4B). This increase persisted for up to 8 days post-treatment (day 17; Figure 4B), demonstrating that PTC299 effectively accelerated platelet recovery in radiation-induced thrombocytopenia. However, PTC299 treatment had a relatively small overall effect on mean platelet volume (MPV) or platelet distribution width (PDW), suggesting minimal effect on platelet size (Figure 4C and D). However, PTC299 treatment may have a potential stimulatory effect on RBC counts, while not affecting white blood cell (WBC) levels (Figures 4E and F), indicating that PTC299 selectively accelerates platelet and erythrocyte recovery with minimal impact on other hematopoietic lineages.

### PTC299 promotes bone marrow megakaryocyte production in mice with radiation-induced thrombocytopenia

**Figure 5.**
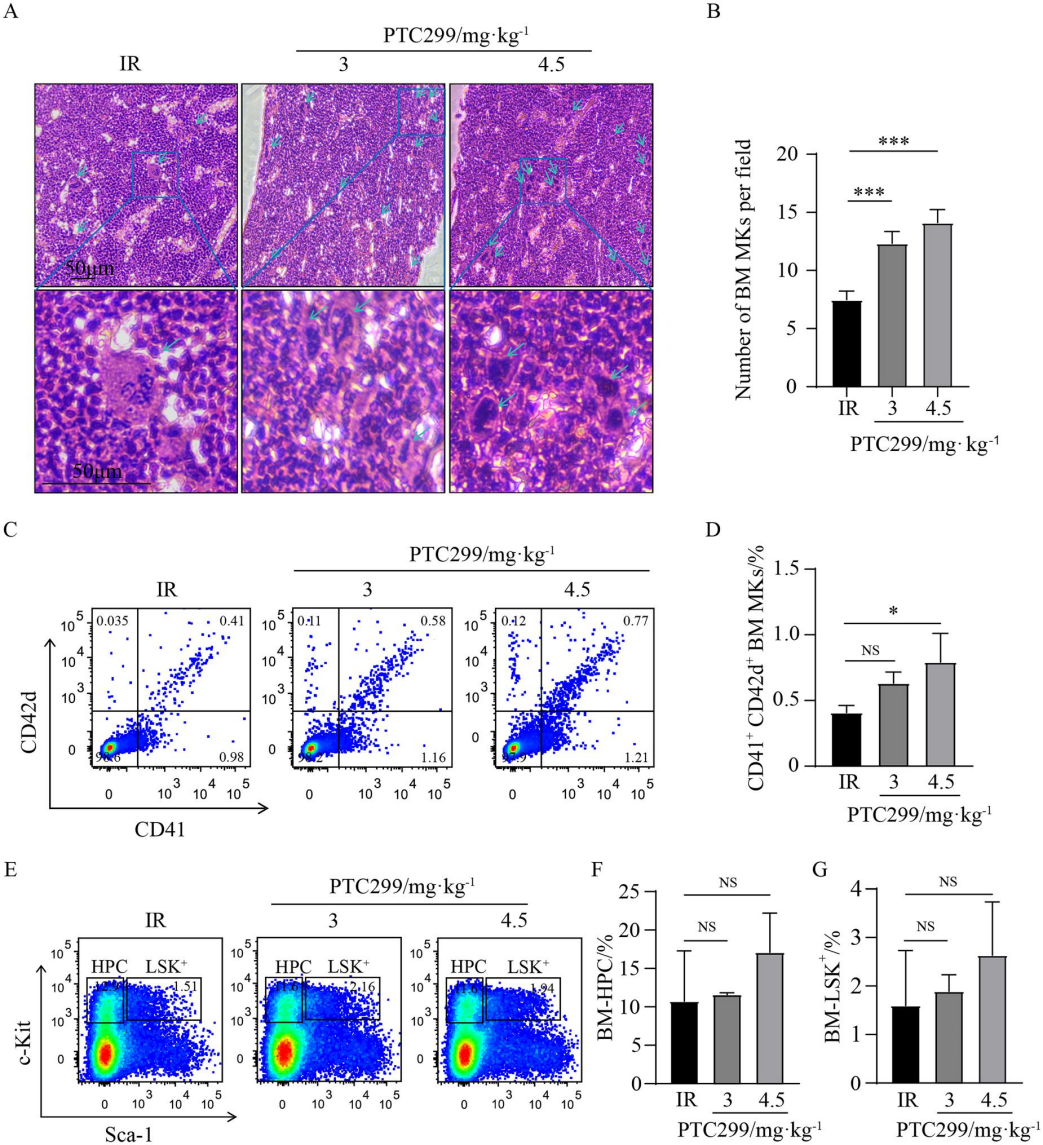
PTC299 promotes megakaryocyte differentiation in the bone marrow of radiation-induced thrombocytopenic mice. (A) Representative HE staining images (20× magnification) of tibia in a mouse model of X-ray-induced thrombocytopenia irradiated at 4.5 Gy and treated with PTC299 (3 or 4.5 mg/kg) or left untreated (IR control). Arrows indicate megakaryocytes. Scale bar: 50 μm. (B) Quantification of the number of megakaryocytes per field of mouse tibia with HE staining (20× magnification) related to (A), in comparison with the control group. One-way ANOVO followed by Dunnett’s *post hoc* test, ***P < .001, *n* = 7. (C) Representative flow cytometry analyses demonstrating the proportion of CD41^+^CD42d^+^ megakaryocytes in mouse bone marrow in the thrombocytopenia mouse model treated with PTC299 (3 or 4.5 mg/kg) or left untreated (IR control). (D) Quantification of the proportion of CD41^+^CD42d^+^ megakaryocytes in the bone marrow of mice in each group related to (C), in comparison with the control group. One-way ANOVO followed by Dunnett’s *post hoc* test, *P < .05, *n* = 3. (E) Representative flow cytometry analyses demonstrating the proportion of hematopoietic stem progenitor (HPC and LSK^+^) cells in mouse bone marrow of the thrombocytopenia mouse model treated with PTC299 (3 or 4.5 mg/kg) or left untreated (IR control). (F) Quantification of the proportion of HPC in mouse bone marrow in each group related to (E), in comparison with the control group. One-way ANOVO followed by Dunnett’s *post hoc* test, *n* = 3. (G) Quantification of the proportion of LSK^+^ cells in mouse bone marrow in each group related to (E), in comparison with the control group. One-way ANOVO followed by Dunnett’s *post hoc* test, *n* = 3.

To further elucidate the effect of PTC299 on megakaryocyte differentiation *in vivo*, we examined bone marrow megakaryocyte numbers using HE staining and flow cytometry. HE staining revealed an increased presence of nucleated cells in the bone marrow of mice treated with 3 and 4.5 mg/kg PTC299 compared to the control group (IR), with a significant rise in megakaryocyte numbers (Figure 5A and B). Consistently, flow cytometry analysis demonstrated a significantly higher proportion of CD41^+^CD42d^+^ megakaryocytes in the PTC299-treated groups than in the control group (Figure 5C and D). These findings indicate that PTC299 administration significantly augments megakaryocyte differentiation in radiation-induced thrombocytopenia mice.

To determine whether PTC299 enhances megakaryocyte production by stimulating the proliferation of hematopoietic stem and progenitor cells (HSPCs), we analyzed the proportion of HSPCs in bone marrow via flow cytometry. The results showed no significant differences between PTC299-treated and control groups (Figure 5E-G), suggesting that PTC299 primarily promotes megakaryocyte production by enhancing HSPC differentiation rather than proliferation.

### PTC299 boosts splenic megakaryocyte production and reduces radiation-induced splenomegaly in thrombocytopenic mice

**Figure 6.**
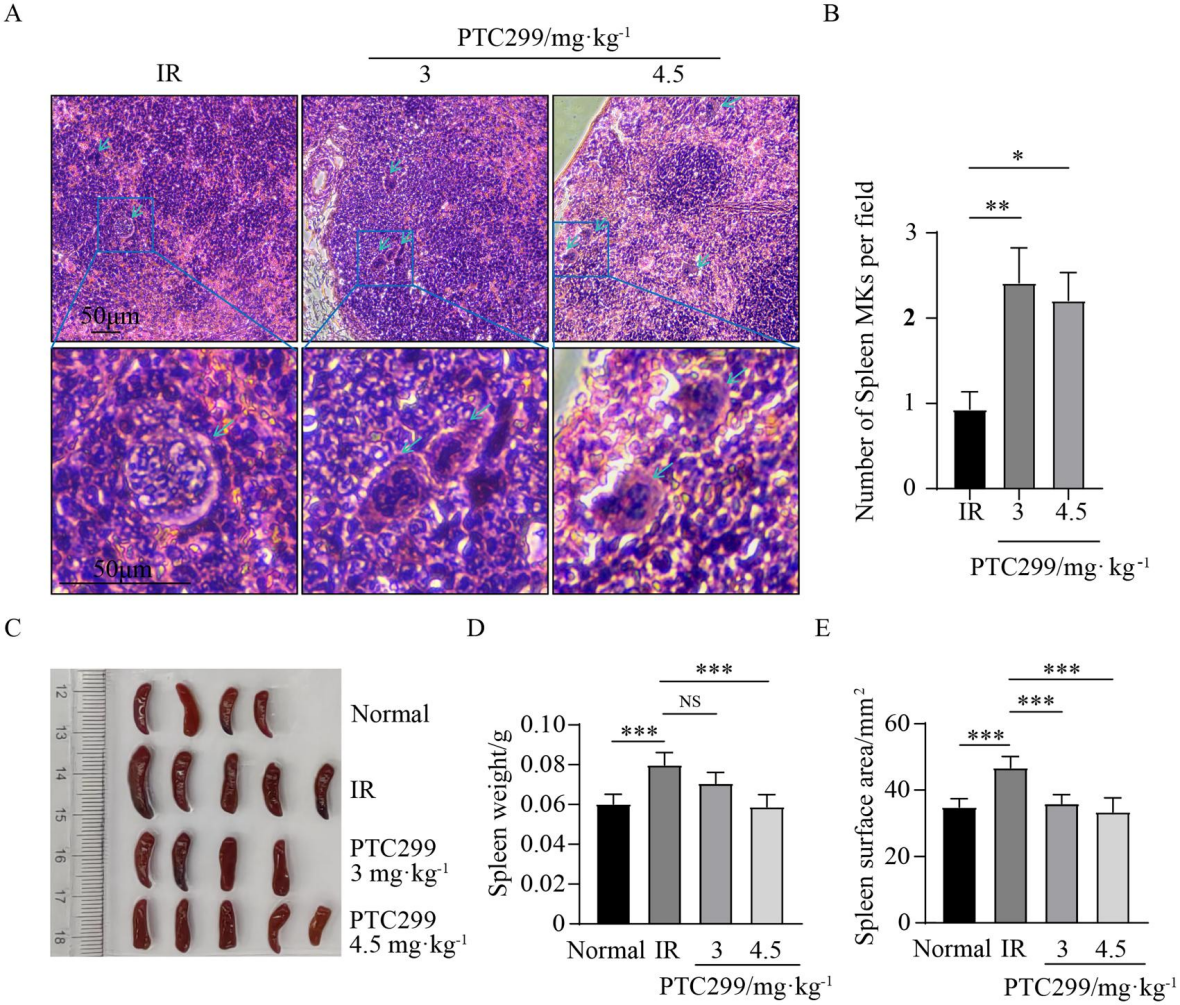
PTC299 enhances spleen megakaryocyte production and reduces spleen enlargement in radiation-induced thrombocytopenic mice. (A) HE staining of the spleens of mice after irradiated with 4.5 Gy of X-rays 13 days (day 13). Arrows indicate megakaryocytes. Scale bar: 50 μm. (B) Quantification showing the number of splenic megakaryocytes per field related to (A), in comparison with the control group. One-way ANOVO followed by Dunnett’s *post hoc* test, *P < .05, **P < .01, *n* = 7. (C) Spleen images from untreated C57BL/6 mice (normal), post-irradiation mice (IR), and mice treated with PTC299 (3 and 4.5 mg/kg) starting on day 7 post-irradiation. (D) Spleen weights of mice for each group related to (C), in comparison with the IR group. One-way ANOVO followed by Dunnett’s *post hoc* test, ***P < .001, *n* ≥4. Outlier was detected by the Grubbs’ method (alpha = 0.01) and removed from the analysis. (E) Spleen surface area for each group related to (C), in comparison with the IR group. One-way ANOVO followed by Dunnett’s *post hoc* test, ***P < .001, *n* ≥4.

The spleen plays a key role as an extramedullary stress hematopoietic organ. To assess the impact of PTC299 on splenic megakaryocyte production, we examined the number of megakaryocytes in splenic tissues using HE staining. The results showed a significant increase in the average number of megakaryocytes per 10x field in the spleens of mice treated with 3 and 4.5 mg/kg PTC299 compared to the control group (Figure 6A and B), indicating that PTC299 enhances splenic megakaryopoiesis in radiation-induced thrombocytopenia *in vivo*. Post-irradiation stress hematopoiesis typically leads to spleen enlargement.^35^ Consistent with this, the spleens of mice in the irradiated group (IR group) were significantly larger than those of untreated C57BL/6 mice 13 days after 4.5 Gy X-ray exposure (Figure 6C). However, the spleen size and weight of mice treated with 3 and 4.5 mg/kg PTC299 after irradiation were significantly smaller than those in the IR group (Figure 6C-E). These findings suggest that PTC299 not only promotes the differentiation of splenic megakaryocytes in radiation-induced thrombocytopenia but also alleviates radiation-induced splenic hyperfunction in mice.

## Discussion

Thrombocytopenia, characterized by an abnormally low platelet count, can result from impaired megakaryocyte function, reduced platelet production, or excessive platelet destruction.^36^ This condition increases the risk of spontaneous bleeding and complicates treatments such as chemotherapy and radiation therapy.^37^ Current therapeutic strategies for thrombocytopenia primarily rely on platelet transfusions and TPO receptor agonists.^38^^,^^39^ While transfusions provide immediate relief, they are limited by donor dependency, high costs, short shelf life, and alloimmune reactions.^40^ Similarly, TPO receptor agonists such as romiplostim and eltrombopag stimulate megakaryopoiesis but are associated with risks including thrombotic complications, drug resistance, and off-target effects.^38^ Given these challenges, alternative approaches to enhance platelet production are urgently needed. *In vitro* platelet generation represents a promising strategy, offering a renewable platelet source for transfusion medicine. However, current *in vitro* systems face limitations, including low yields, scalability issues, and high production costs.^41^^,^^42^ Developing novel pharmacological agents that stimulate megakaryopoiesis and thrombopoiesis could revolutionize thrombocytopenia management and support large-scale platelet manufacturing.

In this study, we identified PTC299 as a novel pro-plateletogenic modulator that enhances megakaryocyte differentiation and platelet production. PTC299 is a potent DHODH inhibitor with previously characterized anti-proliferative effects in cancer cells.^43^ However, its role in megakaryopoiesis and thrombopoiesis has not been explored. We first employed a culture system to differentiate fetal liver nucleated cells into megakaryocytes and conducted a small-molecule screening, identifying PTC299 as a candidate compound that promotes megakaryocyte generation. To further validate its effects, we established an *in vitro* differentiation system using hematopoietic stem/progenitor cells (HSPCs) isolated from embryonic day 15.5 (E15.5) mice. Treatment with PTC299 at varying concentrations led to a significant increase in the percentage of CD41^+^CD42d^+^ megakaryocytes, key surface markers of megakaryocyte differentiation. In addition, ploidy analysis revealed that PTC299-treated megakaryocytes exhibited higher ploidy levels, indicating enhanced endomitosis and polyploidization—critical processes for megakaryocyte maturation. However, the magnitude of PTC299’s effects on megakaryocyte output and ploidy is modest, it appears to promote megakaryocyte differentiation in a synergistic manner in multiple ways rather than having a dramatic effect on any single aspect. Immunofluorescence staining and flow cytometry analyses confirmed that PTC299 significantly promoted platelet production from fetal liver progenitor-derived megakaryocytes and primary megakaryocytes, demonstrating its ability to enhance thrombopoiesis *in vitro*. These findings establish a previously unrecognized role for PTC299 in megakaryocyte and platelet generation, offering a promising pharmacological approach for *in vitro* platelet production.

Furthermore, to evaluated the effect of PTC299 on platelet production of human derived culture systems, we established a human induced pluripotent stem cell (hiPSC)-derived megakaryocyte differentiation and platelet generation system. However, PTC299 treatment did not clearly promote platelet output. The discrepancy between the mouse liver HSPC-derived megakaryocyte system and the hiPSC-based model may reflect inherent limitations of current hiPSC-derived megakaryopoiesis platforms. Indeed, hPSC-derived HSPCs are known to differ from those isolated from *in vivo* human sources.^44–46^ Differences in the optimal concentration or treatment window between human and murine megakaryopoiesis, as well as other undefined factors, may also contribute. Therefore, the effects of PTC299 on human megakaryopoiesis require further clarification.

To evaluate the therapeutic potential of PTC299 *in vivo*, we utilized a mouse model of radiation-induced thrombocytopenia, a clinically relevant condition caused by ionizing radiation exposure. Radiation therapy, commonly used in cancer treatment, can lead to severe bone marrow suppression, resulting in thrombocytopenia due to impaired megakaryocyte differentiation and increased bone marrow cell apoptosis.^47^ In our study, 8-week-old C57BL/6 mice were exposed to 4.5 Gy X-ray irradiation, which significantly reduced circulating platelet levels. Administration of 4.5 mg/kg PTC299 accelerated platelet recovery, with treated mice displaying a faster rebound in platelet counts compared to vehicle-treated controls and 3 mg/kg PTC299 administration. However, the relatively late administration time (day 9 post-irradiation) and lower concentration may have resulted in a less dramatic increase in platelet count. Additionally, bone marrow histological analysis revealed an increase in megakaryocyte numbers in PTC299-treated mice, suggesting that the compound enhances megakaryocyte differentiation and survival following radiation-induced damage. Notably, PTC299 also promoted extramedullary megakaryopoiesis, as evidenced by an increase in splenic megakaryocytes. This finding suggests that PTC299 not only supports bone marrow-derived thrombopoiesis but also stimulates compensatory platelet production in the spleen, a mechanism often observed during hematopoietic stress.

An essential consideration for any thrombopoietic agent is its safety profile. In our study, PTC299 treatment did not cause noticeable adverse effects, as assessed by body weight monitoring, appetite, and general health status. Furthermore, complete blood count analysis indicated that PTC299 selectively accelerated platelet recovery without significantly affecting WBC counts, suggesting minimal off-target effects on leukopoiesis. Importantly, flow cytometry analysis of bone marrow cells revealed that the proportion of hematopoietic stem/progenitor cells remained unchanged following PTC299 treatment, indicating that the compound does not disrupt early hematopoiesis. Additionally, radiation-induced thrombocytopenic mice exhibited significant splenomegaly due to hematopoietic compensation. PTC299 administration significantly reduced splenic enlargement, suggesting that it may mitigate radiation-induced hematopoietic stress and improve bone marrow function. However, PTC299 also exhibited a modest effect on erythrocyte recovery suggesting a potential area for future investigation, with further long-term studies required to comprehensively evaluate the safety and potential off-target effects of PTC299 *in vivo*.

How does PTC299 enhance megakaryocyte differentiation and platelet production? PTC299 is a potent inhibitor of DHODH and an oral VEGFA mRNA translation inhibitor, selectively blocking VEGF protein synthesis at the post-transcriptional level.^48^ DHODH is a key enzyme in de novo pyrimidine biosynthesis, which is essential for cell proliferation and differentiation.^49^^,^^50^ Inhibition of DHODH has been reported to modulate hematopoietic differentiation by affecting nucleotide availability, mitochondrial function, and oxidative stress responses.^51^^,^^52^ Our study suggests that PTC299 may influence megakaryopoiesis through DHODH inhibition without affecting mitochondrial depolarization or reactive oxygen species (ROS) generation, potentially promoting differentiation over proliferation by shifting metabolic states within megakaryocyte precursors. While previous studies have demonstrated that VEGF promotes megakaryocyte maturation, and its blockade via Flk inhibits megakaryocyte polyploidization, our study found that PTC299 treatment did not affect VEGFA secretion and moderately enhances megakaryocyte polyploidization in an i*n vitro* HSPC-based differentiation system. This apparent contradiction suggests that PTC299 may exert its effects through mechanisms independent of VEGF, potentially involving alternative molecular targets that regulate megakaryopoiesis and thrombopoiesis. Further research is needed to delineate the precise pathways through which PTC299 enhances megakaryocyte differentiation and platelet production.

## Conclusion

We investigated mitochondria-targeted compounds for their potential to enhance megakaryocyte and platelet production. The results demonstrate that PTC299, a DHODH and VEGFA mRNA translation inhibitor, effectively enhances megakaryocyte differentiation and platelet production *in vitro*, highlighting its potential for supporting large-scale platelet manufacturing. Additionally, PTC299 promotes platelet recovery and mitigates hematopoietic stress in a mouse model of radiation-induced thrombocytopenia, underscoring its potential as a therapeutic agent for thrombocytopenia. Given its favorable safety profile, PTC299 represents a promising candidate for further preclinical development. Future studies should explore its role in human megakaryocytes and platelet production, investigate its molecular mechanisms in greater detail, investigate its efficacy in additional thrombocytopenia models, and assess its long-term effects on hematopoiesis. By advancing our understanding of thrombopoietic regulation and identifying novel potential pharmacological interventions, this study will provide new perspectives into the development of innovative therapies for thrombocytopenia.

## Supplementary Material

szag035_Supplementary_Data

## Data Availability

The data underlying this article are available in the article and in its online supplementary material.
