# Supplementary material for: The mitochondria-targeting compound PTC299 enhances megakaryocyte and platelet production
Source: Stem Cells Transl Med. 2026 Jun 27;15(7):szag035. doi: 10.1093/stcltm/szag035 (PMC13311664; doi:10.1093/stcltm/szag035)
Supplement: szag035_Supplementary_Data [file szag035_supplementary_data.zip › Table S1 The information of examined small molecule.docx]

Table S1 The information of examined small molecules.

| Small molecule | Target | Concentration |
| --- | --- | --- |
| PTC299 | Dihydroorotate Dehydrogenase; DNA/RNA Synthesis; VEGFR | 1 μM |
| DSM502 | Dihydroorotate Dehydrogenase; Parasite | 4 nM |
| VER-246608 | PDHK | 40 nM |
| CCCP | Apoptosis; Bacterial; IFNAR; Mitochondrial Metabolism; STING | 1 μM |
| R-AGN1135 | Apoptosis; Autophagy; Monoamine Oxidase | 100 nM |
| Hesperidin | Apoptosis; Autophagy; Endogenous Metabolite; Reactive Oxygen Species | 1 μM |
| Harmane | Adrenergic Receptor; Imidazoline Receptor; Monoamine Oxidase | 5 μM |
| UK-5099 | Mitochondrial Metabolism | 50 nM |
| KL1333 | Mitochondrial Metabolism | 200 nM |
| DS-1001b | Isocitrate Dehydrogenase (IDH) | 1 μM |
| Toloxatone | Monoamine Oxidase | 200 nM |
| Camalexin | Bacterial; Fungal; Reactive Oxygen Species | 200 nM |
| ML390 | Dihydroorotate Dehydrogenase | 50 nM |
| AZD7545 | PDHK | 180 nM |
| Mito-apocynin | Mitochondrial Metabolism | 50 nM |
| PAT-1251 | Monoamine Oxidase | 4 μM |
| BPTES | Glutaminase | 1 μM |
| Brofaromine | Monoamine Oxidase | 1 μM |
| FCCP | Mitochondrial Metabolism | 200 nM |
| J14 | Reactive Oxygen Species | 20 μM |
| Lexibulin | Apoptosis; Microtubule/Tubulin; Reactive Oxygen Species | 50 nM |
| Mito-LND | Autophagy; Mitochondrial Metabolism; Reactive Oxygen Species | 250 nM |
| Elesclomol | Apoptosis; Reactive Oxygen Species | 50 nM |
