# Supplementary material for: The mitochondria-targeting compound PTC299 enhances megakaryocyte and platelet production
Source: Stem Cells Transl Med. 2026 Jun 27;15(7):szag035. doi: 10.1093/stcltm/szag035 (PMC13311664; doi:10.1093/stcltm/szag035)
Supplement: szag035_Supplementary_Data [file szag035_supplementary_data.zip › Supplementary information_final.docx]

**Supplemental Information**

**The mitochondria-targeting compound PTC299 enhances megakaryocyte and platelet production**

Xiaoli Wang^a, b, c, d, 1^, Meijuan Xia^b, c, 1^, Yifei Cai^b, c, 1^, Ziqi Huo^b, c, 1^, Yao Zhong^b, c^, Pei Su^b, c^, Cuicui Liu^b, c^, Hongtao Wang^b, c^, Xiangchun Shen^a^, Jianwei Xu^a, d, *^, Fei Wang^e, *^, Jiaxi Zhou^a, b, c, *^

^a^School of Pharmacy, Guizhou Medical University, Guiyang 550025, China

^b^State Key Laboratory of Experimental Hematology, National Clinical Research Center for Blood Diseases, Haihe Laboratory of Cell Ecosystem, Institute of Hematology & Blood Diseases Hospital, Chinese Academy of Medical Sciences & Peking Union Medical College, Tianjin 300020, China

^c^Tianjin Institutes of Health Science, Tianjin 301600, China

^d^Center for Tissue Engineering and Stem Cell Research, Guizhou Medical University, Guiyang 551113, China.

^e^HaemoCure Inc, Tianjin 300459, China

^1^ These authors contributed equally to this work

**^*^ Corresponding authors:** [363912577@qq.com,](mailto:363912577@qq.com,) [feiwang975@gmail.com,](mailto:feiwang975@gmail.com,) [zhoujx@ihcams.ac.cn](mailto:zhoujx@ihcams.ac.cn)

**Supplemental figure legends**

**Figure S1. The identification of mitochondrial-targeted compounds that affect mouse megakaryocyte production.**

1. Representative photomicrographs of megakaryocyte differentiation on day 3 after the treatment of fetal liver cells with different concentrations of PTC299 (0.05 μM, 0.2 μM, 1 μM, 5 μM) (scale bar = 100 μM).
2. Quantification of the number of large cells (>30 μm) per field on day 3 after treatment with different concentrations of PTC299 (0.05 μM, 0.2 μM, 1 μM, 5 μM), in comparison with the control (vehicle only) group. One-way ANOVO followed by Dunnett’s post hoc test, NS, not significant, ****P* <0.001.
3. Representative flow plots of fetal liver cells treated with different concentrations of PTC299 (0.05 μM, 0.2 μM, 1 μM, 5 μM) on day 3.

D. Percentage of CD41^+^ cells on day 3 after treatment of fetal liver cells with different concentrations of PTC299 (0.05 μM, 0.2 μM, 1 μM, 5 μM), in comparison with the control. One-way ANOVO followed by Dunnett’s post hoc test, NS, not significant, ***P* <0.01, ****P* <0.001.

**Figure S2. PTC299 promotes megakaryocyte differentiation and maturation of fetal liver HSPCs**

A. Percentages and numbers of live cells on day 3 after treatment of HSPCs with different concentrations of PTC299 (0.5, 1, and 2 μM), compared with the control. One-way ANOVO followed by Dunnett’s post hoc test, NS, not significant, n = 4.

B. Percentages of JC-1 monomers in MKs, ROS⁺ MKs, and Annexin V⁺PI⁻/ Annexin V⁺PI+MKs on day 3 after treatment of HSPCs with PTC299 (1 μM), compared with the control. Unpaired t test, NS, not significant, n = 4.

C. Concentration (pg/mL) of VEGFA protein on day 3 after treatment of HSPCs with PTC299 (1 μM), compared with the control. Unpaired t test, NS, not significant, n = 3.

D. Percentages of CD41⁺CD42d⁺ cells on day 3 after treatment of HSPCs with different concentrations of Teriflunomide (0.5, 1, and 2 μM) or Brequinar (10, 100, and 200 nM), compared with the control. One-way ANOVO followed by Dunnett’s post hoc test, NS, not significant; **P* < 0.05, n = 4.

**Figure S3. PTC299 promotes platelet production of mouse fetal liver HSPC derived megakaryocytes *in vitro.***

1. Immunofluorescence images of thrombopoiesis on day 4 after treatment with different concentrations of PTC299 (0.5 μM, 1 μM, and 2 μM); scale bar = 100 μm.

B. Quantification of the proportion of CD41^+^CD61^+^ and CD42d^+^CD61^+^ platelets generated by fetal liver HSPC derived megakaryocytes on day 5 after treatment with different concentrations of PTC299 (0.5 μM, 1 μM, and 2 μM), in comparison with the control. One-way ANOVO followed by Dunnett’s post hoc test, NS, not significant, * *P* < 0.05, ** *P* < 0.01, n≥3.

C. Number of CD41^+^CD61^+^ and CD42d^+^CD61^+^ platelets produced by fetal liver HSPC derived megakaryocytes on day 5 after treatment with different concentrations of PTC299 (0.5 μM, 1 μM and 2 μM), in comparison with the control. One-way ANOVO followed by Dunnett’s post hoc test, NS, not significant, * *P* < 0.05, ** *P* < 0.01, n≥3.

D. Quantification of the proportion of CD41^+^CD61^+^ and CD42d^+^CD61^+^ platelets generated by fetal liver HSPC derived megakaryocytes on day 5 after treatment with different concentrations of Teriflunomide (0.5, 1, and 2 μM) or Brequinar (10, 100, and 200 nM), in comparison with the control. One-way ANOVO followed by Dunnett’s post hoc test, NS, not significant, * *P* < 0.05, n≥3.

E. Number of CD41^+^CD61^+^ and CD42d^+^CD61^+^ platelets produced by fetal liver HSPC derived megakaryocytes on day 5 after treatment with different concentrations of Teriflunomide (0.5, 1, and 2 μM) or Brequinar (10, 100, and 200 nM), in comparison with the control. One-way ANOVO followed by Dunnett’s post hoc test, NS, not significant, n≥3.

F. Fold change in the proportion of CD41⁺CD61⁺ and CD42d⁺CD61⁺ platelets generated from fetal liver HSPC-derived megakaryocytes on day 5 after treatment with PTC299 (1 μM), Teriflunomide (0.5 μM), or Brequinar (10 nM), compared with the control. One-way ANOVO followed by Dunnett’s post hoc test, NS, not significant; ***P* < 0.01, n≥3.

G. Fold change in the number of CD41⁺CD61⁺ and CD42d⁺CD61⁺ platelets produced by fetal liver HSPC-derived megakaryocytes on day 5 after treatment with PTC299 (1 μM), Teriflunomide (0.5 μM), or Brequinar (10 nM), compared with the control. One-way ANOVO followed by Dunnett’s post hoc test, NS, not significant, n≥3.

**Figure S4. PTC299 did not clearly enhance platelet output in hPSC-based system.**

A. Quantification of the proportion of CD41^+^ platelets derived from hPSCs on day 9 after treatment with 1 μM PTC299, in comparison with the control. Unpaired t test, **P* < 0.05.

B. The number of CD41^+^ platelets derived from hPSCs on day 9 after treatment with 1 μM PTC299, in comparison with the control. Unpaired t test, NS, not significant.
