# Supplementary figures and images for: The mitochondria-targeting compound PTC299 enhances megakaryocyte and platelet production
Source: Stem Cells Transl Med. 2026 Jun 27;15(7):szag035. doi: 10.1093/stcltm/szag035 (PMC13311664; doi:10.1093/stcltm/szag035)

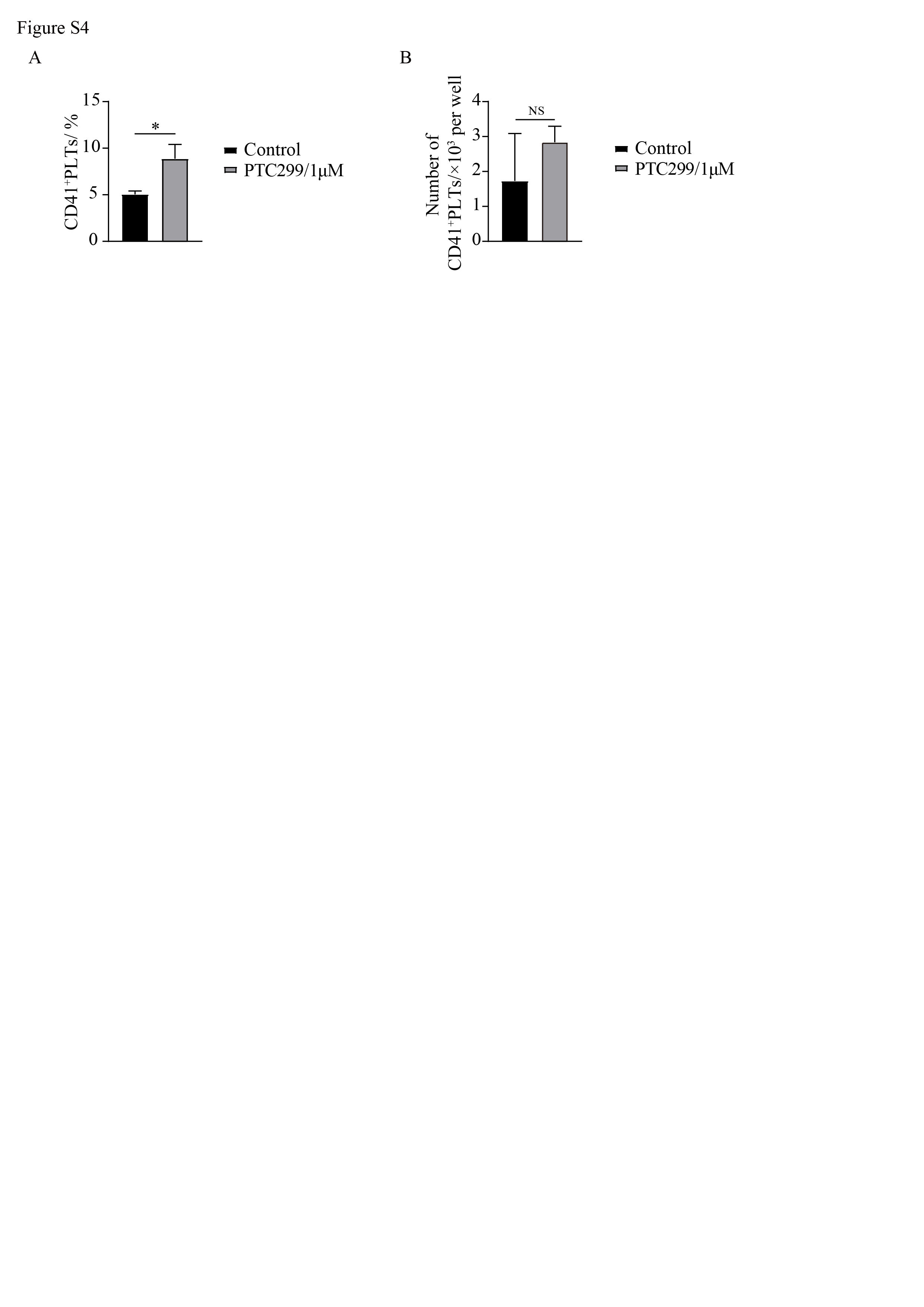

Supplement: szag035_Supplementary_Data [file szag035_supplementary_data.zip › Figure S4-R2.tif]

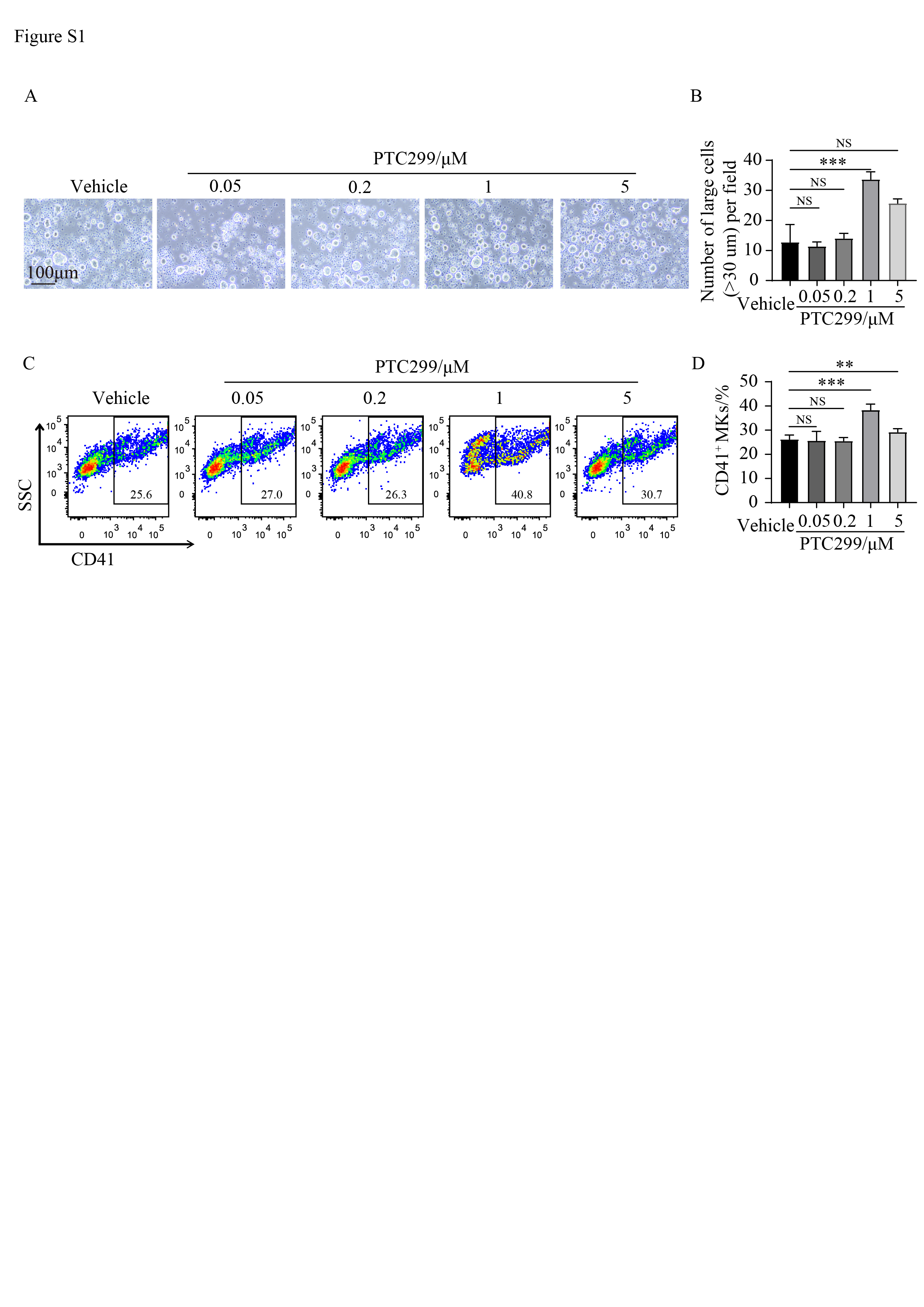

Supplement: szag035_Supplementary_Data [file szag035_supplementary_data.zip › Figure S1-R2.tif]

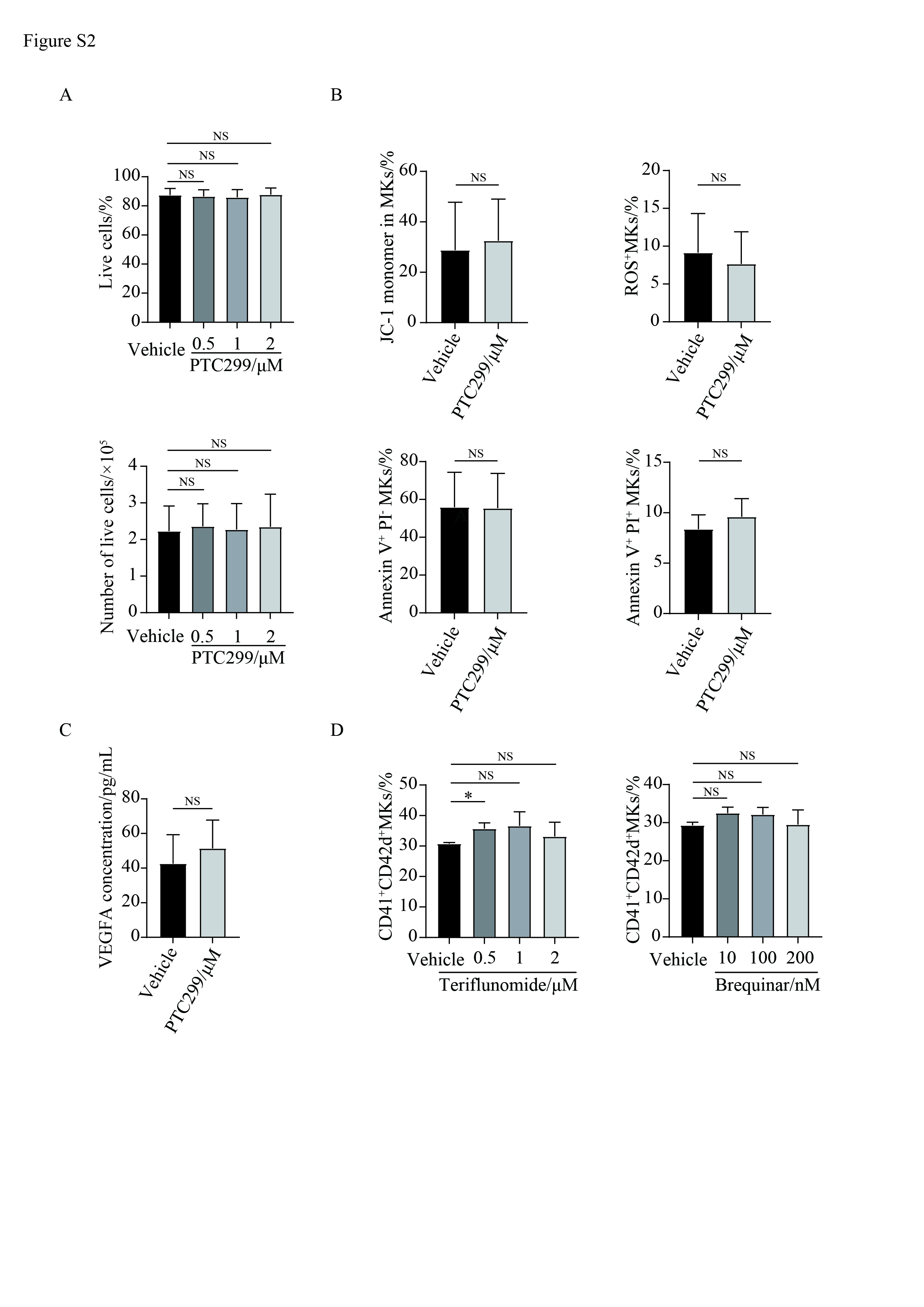

Supplement: szag035_Supplementary_Data [file szag035_supplementary_data.zip › Figure S2-R2.tif]

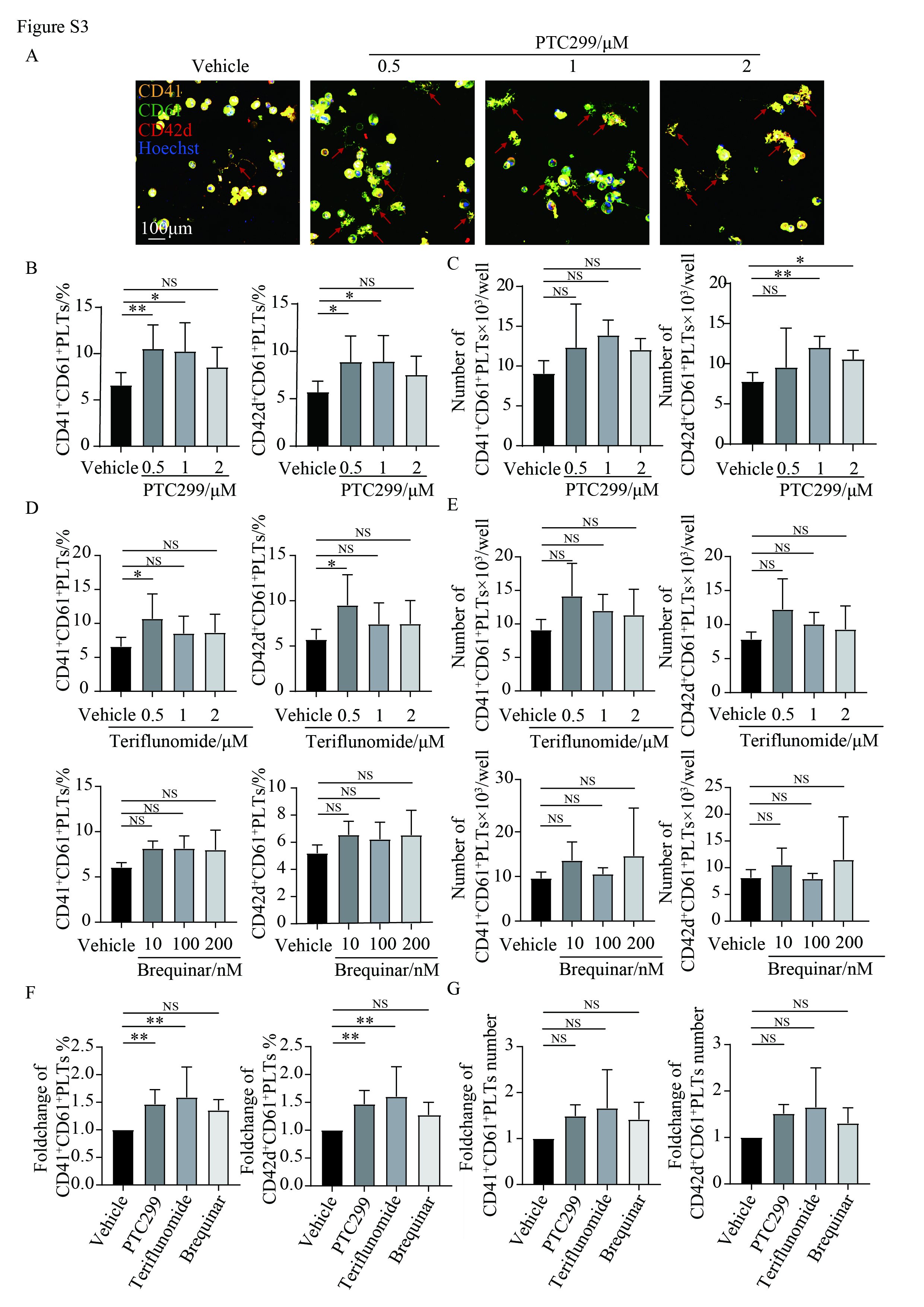

Supplement: szag035_Supplementary_Data [file szag035_supplementary_data.zip › Figure S3-R2.tif]
